# Global Research on Coronaviruses: An R Package

**DOI:** 10.2196/19615

**Published:** 2020-08-11

**Authors:** Thierry Warin

**Affiliations:** 1 HEC Montreal Montreal, QC Canada

**Keywords:** COVID-19, SARS-CoV-2, coronavirus, R package, bibliometric, virus, infectious disease, reference, informatics

## Abstract

**Background:**

In these trying times, we developed an R package about bibliographic references on coronaviruses. Working with reproducible research principles based on open science, disseminating scientific information, providing easy access to scientific production on this particular issue, and offering a rapid integration in researchers’ workflows may help save time in this race against the virus, notably in terms of public health.

**Objective:**

The goal is to simplify the workflow of interested researchers, with multidisciplinary research in mind. With more than 60,500 medical bibliographic references at the time of publication, this package is among the largest about coronaviruses.

**Methods:**

This package could be of interest to epidemiologists, researchers in scientometrics, biostatisticians, as well as data scientists broadly defined. This package collects references from PubMed and organizes the data in a data frame. We then built functions to sort through this collection of references. Researchers can also integrate the data into their pipeline and implement them in R within their code libraries.

**Results:**

We provide a short use case in this paper based on a bibliometric analysis of the references made available by this package. Classification techniques can also be used to go through the large volume of references and allow researchers to save time on this part of their research. Network analysis can be used to filter the data set. Text mining techniques can also help researchers calculate similarity indices and help them focus on the parts of the literature that are relevant for their research.

**Conclusions:**

This package aims at accelerating research on coronaviruses. Epidemiologists can integrate this package into their workflow. It is also possible to add a machine learning layer on top of this package to model the latest advances in research about coronaviruses, as we update this package daily. It is also the only one of this size, to the best of our knowledge, to be built in the R language.

## Introduction

The coronavirus disease (COVID-19) outbreak finds its roots in Wuhan with potential first observations in late 2019 [1]. On December 30, 2019, clusters of cases of pneumonia of unknown origin were reported to the China National Health Commission. On January 7, 2020, a novel coronavirus (2019-nCoV) was isolated. Two previous outbreaks have taken place since the year 2000 involving coronaviruses: (1) severe acute respiratory syndrome–related coronavirus (SARS-CoV) and (2) Middle East respiratory syndrome–related coronavirus (MERS-CoV) [2].

In the context of the global propagation of the virus, numerous initiatives have occurred mobilizing our current global technological infrastructure: (1) the internet and server farms; (2) the convergence in coding languages; and (3) the use of data, structured and unstructured.

First, the internet is used for exchanges between universities, research laboratories, or political leaders to cite a few examples. Second, the convergence in coding languages, in particular functional languages such as R (R Foundation for Statistical Computing), Python (Python Software Foundation), or Julia, has helped facilitate the communications between researchers. Reproducible research has accelerated in these past few years, with principles such as open science, open data, and open code. The use of data has been amplified by the development of new methodologies within the field of artificial intelligence (AI), allowing researchers to generate and analyze structured and unstructured data in a supervised way, as well as in a semi- or unsupervised way [3]. Data initiatives have flourished across the world, collecting firsthand data, aggregating various data sets, and developing simulation-based models. The Johns Hopkins University of Medicine has made a great effort in terms of data visualizations, which has disseminated throughout the world [4]. In doing so, it has undoubtedly helped to raise citizens’ awareness, helped policy makers inform their population and avoid some potential fake news, or helped correct some misconceptions or confusion. The Johns Hopkins initiative has been followed by several others across the world, creating a large variety and diversity of data sets. This creation process allows researchers to benefit from different levels of granularity when it comes to the data dimensions such as geography, indicators, or methodologies; the open science characteristic is also an interesting aspect of the current research contributions [1].

With better access to these new technologies and methodologies, the breadth of expertise is more extensive; epidemiologists are leading the core of the research, but data scientists, biostatisticians, researchers in humanities, or social scientists can contribute and leverage their domain expertise using converging methodologies such as decision trees, text mining, or network analysis [5].

It is with these hypotheses in mind that we propose to replicate the spirit of the Allen Institute for AI initiative and to design an R package, whose main objective is to integrate easily in a researcher's workflow. This package is named EpiBibR.

EpiBibR stands for an “epidemiology-based bibliography for R.” The R package is under the Massachusetts Institute of Technology license and, as such, is a free resource based on the open science principles (reproducible research, open data, and open code). The resource may be used by researchers whose domain is scientometrics but also by researchers from other disciplines. For instance, the scientific community in AI and data science may use this package to accelerate new research insights about COVID-19. The package follows the methodology put in place by the Allen Institute and its partners [6] to create the CORD-19 data set with some differences. The latter is accessible through downloads of subsets or a representational state transfer application programming interface. The data provide essential information such as authors, methods, data, and citations to make it easier for researchers to find relevant contributions to their research questions. Our package proposes 22 features for the 60,500 references (on June 26, 2020), and access to the data has been made as easy as possible to integrate efficiently in almost any researcher's pipeline.

Through this package, a researcher can connect the data to her research protocol based on the R language. With this workflow in mind, a researcher can save time on collecting data and can use an accessible language to perform complex analytical tasks, for instance, be it in R or Python. Indeed, it is usual that researchers use multiple languages (functional or not) to produce specific outputs. This workflow opens these data to analyses from the most extensive spectrum of potential options, enhancing multidisciplinary approaches applied to these data (biostatistics, bibliometrics, and text mining, among others).

The goal of this package in this emergency context is to limit the references to the medical domain (here the PubMed repository) but to then leverage the methodologies used across different disciplines. As we will address this point later, a further extension could be to add references from other disciplines to not only benefit from the wealth of methodologies but also their theories and concepts. For instance, to assess the spread of the disease, the literature—and theories—from researchers in demography would certainly be relevant.

## Methods

### Motivation

Across the world, a couple of initiatives have emerged whose goals are primarily to provide access to medical references. The main objective is to disseminate, as much as possible, the extensive research that has been done in the past (and recent history) to save some time and to improve the efficiency of further investigation. Research processes need to be efficient, and the time spent to perform this research needs to be relevant in this emergency. In addition, by proposing a (as much as possible) comprehensive data set of medical references on the coronavirus topic, the wisdom of crowds principle may play a role. A broader community beyond university researchers may use it and help shorten the time to the vaccine production. Researchers from pharmaceutical companies or other organizations may tap into these data to fine-tune their research and research processes.

A nonexhaustive list of the current bibliographic packages comprises the LitCovid data set from the National Library of Medicine (NLM) [7], the World Health Organization (WHO) data set [8], the “COVID-19 Research Articles Downloadable Database” from the Centers for Disease Control and Prevention (CDC) - Stephen B. Thacker CDC Library [9], and the “COVID-19 Open Research Dataset” by the Allen Institute for AI and their partners [6]. All these resources are essential and serve various complementary purposes. They are disseminated to their respective channels (ie, to their respective audiences). They are tailored to their specific needs. The LitCovid data set comprises 6530 references and can be downloaded from the United States NLM’s website in a format that suits bibliographic software. It deals primarily with research about 2019-nCoV. The WHO’s data set has around 9663 references, also specifically on COVID-19. The CDC’s database is proposed in a software format as well (Microsoft Excel [Microsoft Corp] and bibliographic software formats) and comprises 17,636 references about COVID-19 and the other coronaviruses. The Allen Institute for AI’s data set proposes over 190,000 references about COVID-19 as well as references about the other coronaviruses. It is accessible through different subsets of the overall database and a dedicated search engine. It also taps into a variety of academic article repositories.

In this context, the contributions made by the EpiBibR package are fourfold. First, with more than 60,500 references, EpiBibR is among the most extensive reference databases and is updated daily. The sheer number of references may be more suitable for a broader audience. Second, EpiBibR collects the data exclusively from PubMed to propose a controlled environment. Third, EpiBibR matches the keywords from the Allen Institute for AI’s database to offer some consistency for researchers. Last, it is an R package and, as such, can be integrated into a workflow a little more efficiently than a file necessitating a specific software. Research teams can install the package in their systems and tap into it without the risks of version issues.

Beyond these four differentiation elements, EpiBibR is not better or worse than any other existing database. It just serves its audience and its purpose, like the other databases. It has not been created to replace a current database but, to the contrary, to complement these databases. We do believe that we need more initiatives in this domain at the world stage to support and integrate all the potential audiences and various workflows across the world. As a result, these initiatives would help accelerate research on coronaviruses overall and COVID-19 in particular.

### Functionality

As previously mentioned, EpiBibR is an R package to access bibliographic information on COVID-19 and other coronaviruses references easily. The package can be found at [10]. The command to install it is remotes::install_github ('warint/'EpiBibR'). We advise making sure the latest version of the package has been installed on each researcher’s system. The installation procedure can be found on the README file of this Github account. A full website with the various functions and examples are accessible from this page as well. The vignette has been created based on this paper to extend the use cases as more data are collected.

The references were collected via PubMed, a free resource that is developed and maintained by the National Center for Biotechnology Information at the United States NLM, located at the National Institutes of Health. PubMed includes over 30 million citations from biomedical literature.

More specifically, to collect our references, we adopted the procedure used by the Allen Institute for AI for their CORD-19 project. We applied a similar query on PubMed (“COVID-19” OR Coronavirus OR “Corona virus” OR “2019-nCoV” OR “SARS-CoV” OR “MERS-CoV” OR “Severe Acute Respiratory Syndrome” OR “Middle East Respiratory Syndrome”) to build our bibliographic data.

To navigate through our data set, EpiBibR relies on a set of search arguments: author, author’s country of origin, keyword in the title, keyword in the abstract, year, and the name of the journal. Each of them can genuinely help scientists and R users to filter references and find the relevant articles.

To simplify the workflow between our package and the research methodologies, the format of our data frame has been designed to integrate with different data pipelines, notably to facilitate the use of the R package Bibliometrix with our data [11].

The package comprises more than 60,500 references and 22 features (see Textbox 1).

**Field tags and their descriptions.**

**AU**

Authors

**TI**

Document title

**AB**

Abstract

**PY**

Year

**DT**

Document type

**MESH**

Medical Subject Headings vocabulary

**TC**

Times cited

**SO**

Publication name (or source)

**J9**

Source abbreviation

**JI**

International Organization for Standardization source abbreviation

**DI**

Digital Object Identifier

**ISSN**

Source code

**VOL**

Volume

**ISSUE**

Issue number

**LT**

Language

**C1**

Author address

**RP**

Reprint address

**ID**

PubMed ID

**DE**

‘Authors’ Keywords

**UT**

Unique Article Identifier

**AU_CO**

‘Author’s country of origin

**DB**

Bibliographic database

EpiBibR allows researchers to search academic references using several arguments: author, author’s country of origin, author + year, keywords in the title, keywords in the abstract, year, and source name. Researchers can also download the entire bibliographic data frame comprised of around 60,500 references with 22 metadata each.

In Textbox 2, we provide the descriptions of the functions available in the R language to collect the relevant information.

**Descriptions of the functions to collect the relevant references.**

**EpiBib_data <- EpiBib_references()**

Download the entire bibliographic data frame

**colson_articles <- EpiBib_author(“Colson”)**

Search all the articles written by Philippe Colson

**canada_articles <- EpiBib_country(“canada”)**

Search by ‘author’s country of origin

**yang2019 <- EpiBib_AU_YE(author = “yang”, year = 2019)**

Search by author and year

**covid_articles <- EpiBib_title(“covid”)**

Search by keywords in the title

**coronavirus_articles <- EpiBib_abstract(“coronavirus”)**

Search by keywords in the abstract

**A2020_articles <- EpiBib_year(2020)**

Search by year

**bio_articles <- EpiBib_source(“bio”)**

Search by source

## Results

In what follows, a use case about how we can use such a data set is proposed. This section is not intended to offer a complete systematic literature review of the 60,500 references. This is the purpose of another article. However, we want to illustrate some powerful techniques that can be applied to this collection of references (for instance, social network analysis) while remaining at a very high level [12].

A systematic literature review consists of mainly four stages: (1) planning, (2) conducting, (3) analysis, and (4) synthesis and reporting. In the first stage, a preliminary study aims to build a corpus of articles citing the most relevant articles in the domain. The second stage is about producing a general review of the main topics used in the corpus. The third stage here is about making a cocitation analysis of the references in each corpus article. The last stage is about proposing a keywords co-occurrence analysis [13].

In our context, we propose a slightly modified perspective on a systematic literature review. The first stage is here at a different scale since we collect not just a few relevant articles to create the corpus but a vast, almost exhaustive, list of articles on a topic. It is worth noticing that the process of data selection consists more of what we define as an “algorithmic systematic literature review,” sometimes also referred to as “automation” [14]. An algorithmic systematic literature review comes with lots of benefits. The proposed modified systematic literature review improves the more classical approach since it does not rely on a manual search and extraction, with the potential biases and limitations it might create. An algorithmic systematic literature review combines the strength of both approaches: the power of big data with the academic soundness of the systematic literature review process. As such, it does not replace the expert’s analysis of the literature. On the contrary, it should be used to augment the expert’s study [3].

The *bibliometrix* package allows a thorough bibliometric analysis using R. Our EpiBibR data have been designed to integrate easily with the *bibliometrix* package. A shiny application is also available, called biblioshiny() [11]. This package has been used extensively for various exercises mobilizing massive amounts of data [15].

**Figure 1:**
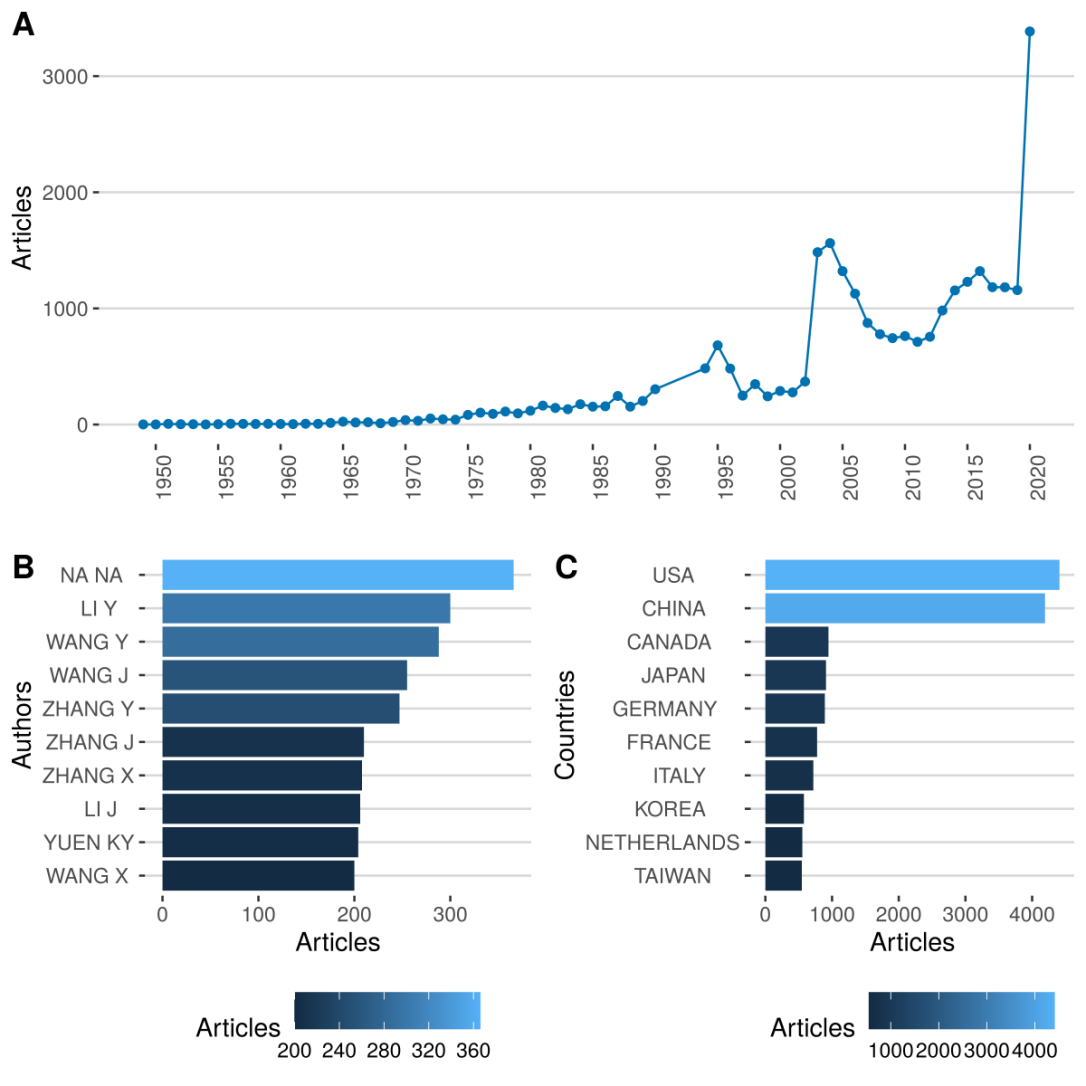
(A) Count of articles, (B) most productive authors, and (C) most productive countries.

Let us first propose a simple count of the references on the coronaviruses literature. In Figure 1, the historical development of research on coronaviruses can be analyzed as having three stages: exploration, initial development, and rapid development in the past year and the current year.

**Figure 2:**
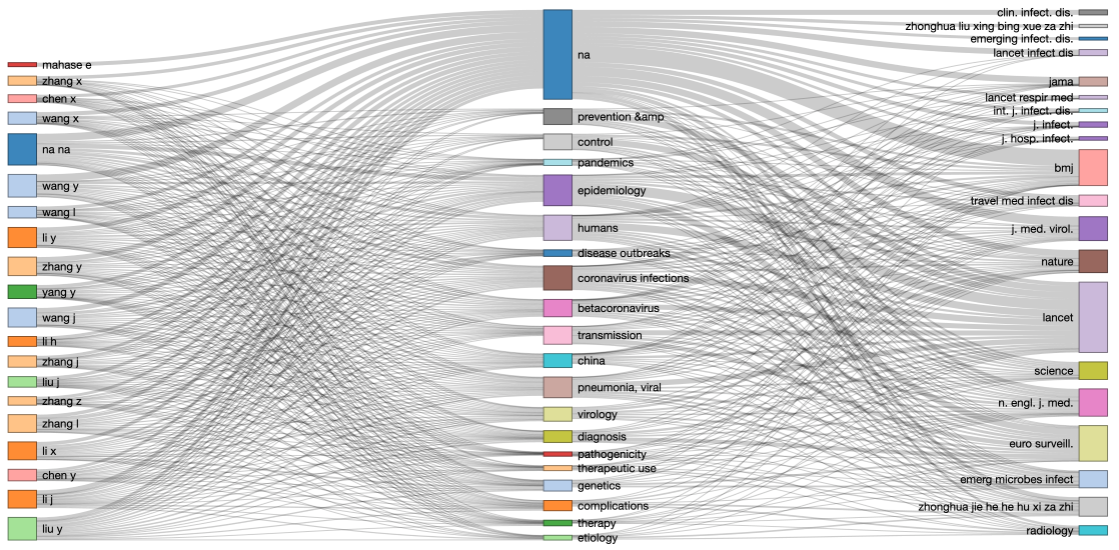
Sankey diagram: authors <> keywords <> sources.

In 2019, a little more than 1000 papers on coronaviruses had been produced, and in the first 5 months of 2020, close to 3400 papers were written on the topic. Those papers from the past 2 years seem to be an interesting, statistically representative sample. In Figure 1, we can also highlight the most productive authors as well as the most productive countries in terms of absolute counts. The most prolific authors provide interesting statistics since it is most likely to proxy research labs. In doing so, we can find which teams are working on which aspect of the coronaviruses. To illustrate our latter point, in Figure 2, we first propose a compelling visualization, called a Sankey diagram. It links authors, keywords, and sources on a connected map. It is the first way to create groups of researchers. The results could be used by policy makers to identify areas of research on this topic.

**Figure 3:**
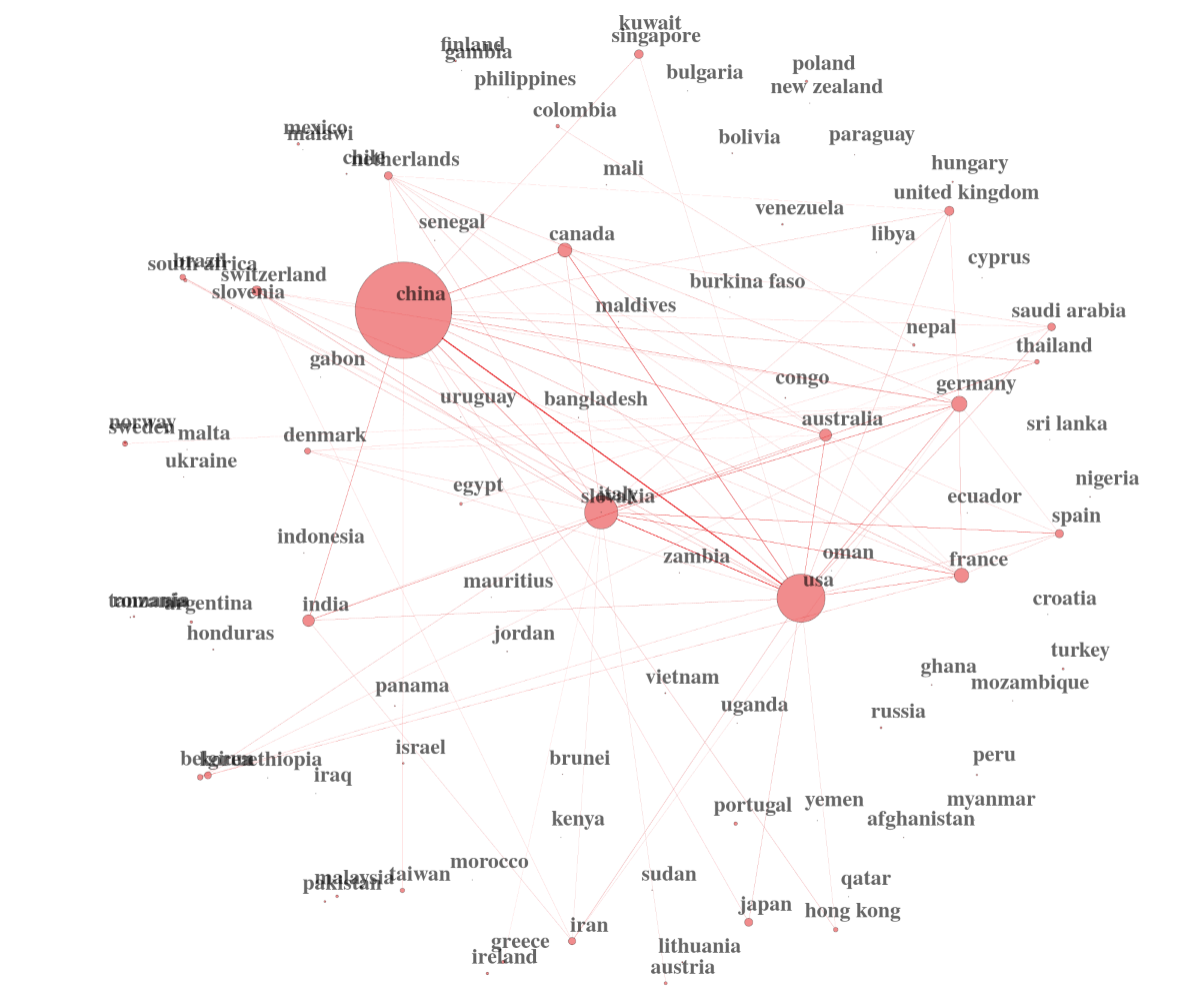
Country collaboration network.

We can also use powerful techniques such as Social Network Theory to find potential clusters of topics, clusters of researchers, and groups of country collaborations. Figure 3 is an example of the latter. The United States and China produce the bulk of the research on coronaviruses.

**Figure 4:**
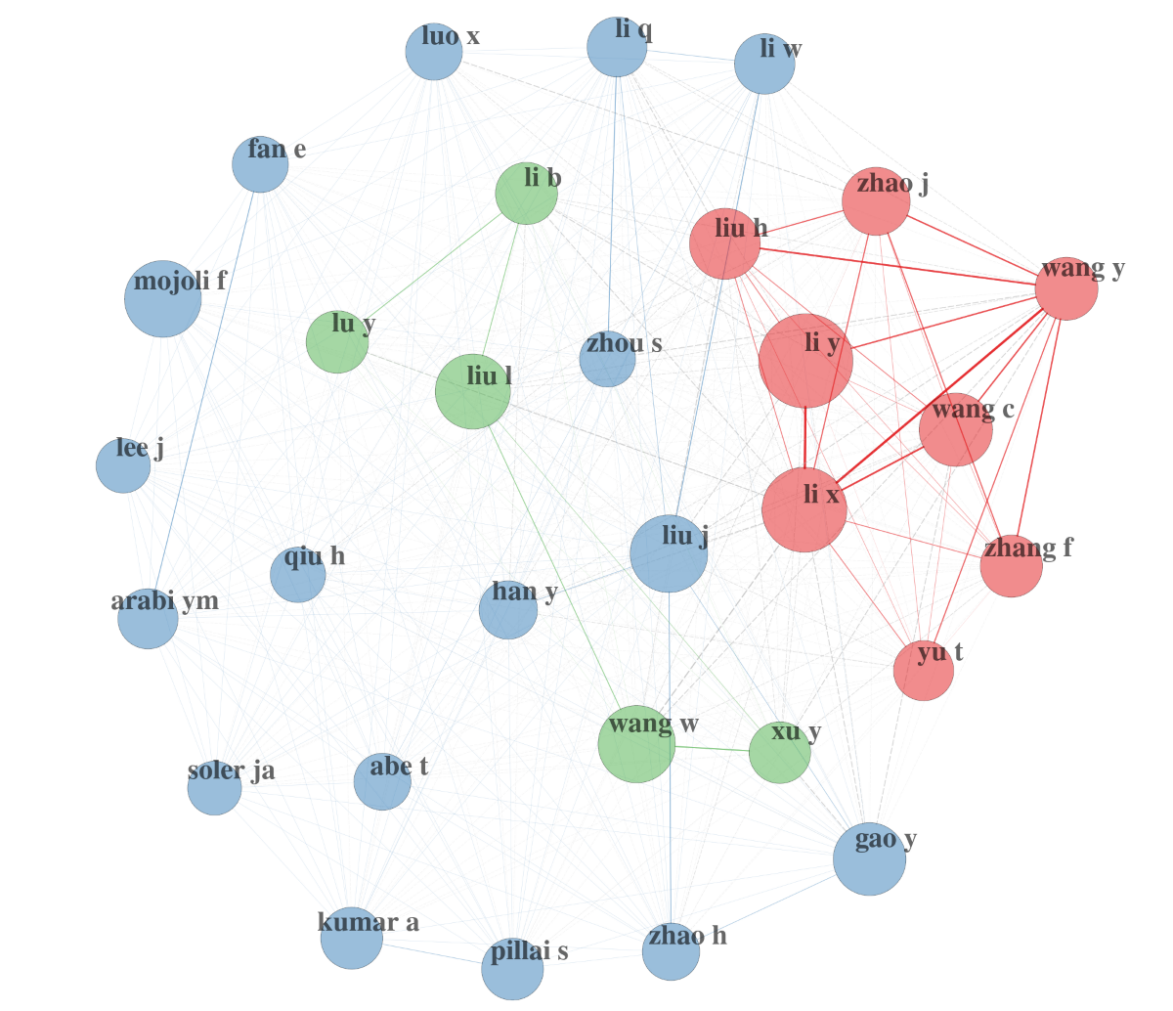
Author collaboration network.

Figure 4 is an illustration of author collaboration networks. As previously mentioned, we remain at a very high level here. However, policy makers or public health officers, for instance, could use these techniques to find more granular networks either just within the 60,500 references or by crossing with other databases. We could even imagine crossing with unstructured data for some specific purposes [16].

**Figure 5:**
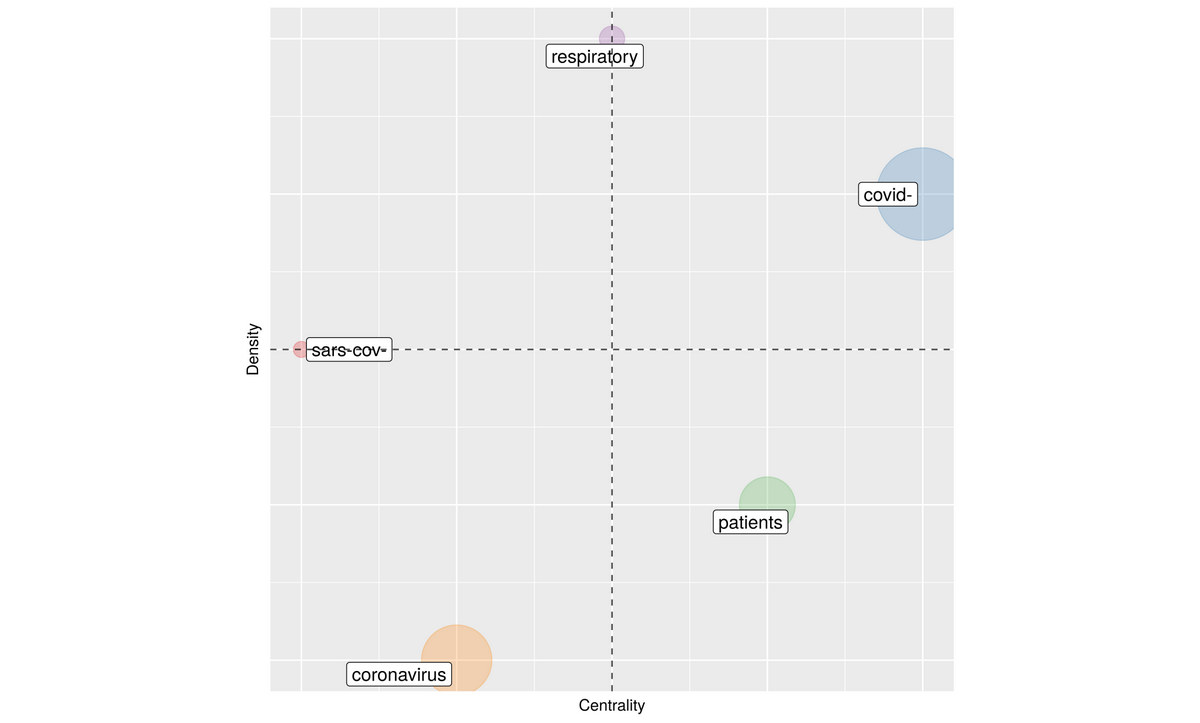
Thematic maps.

Figure 5 is about finding clusters of topics. This technique can be applied to subsamples of the 60,500 references to provide a more granular analysis.

**Figure 6:**
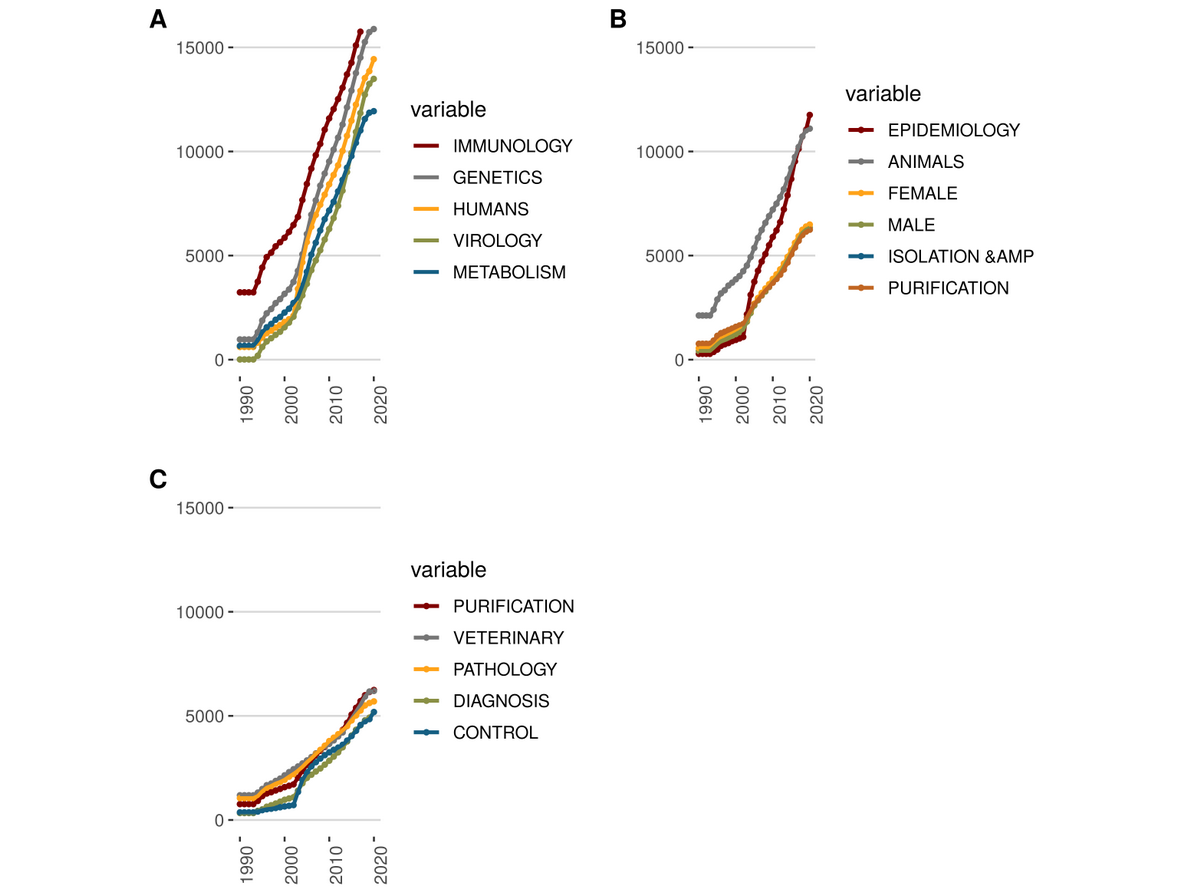
Author's keywords usage evolution over time.

Figure 6 proposes indeed to go further on the topic dimension. For instance, we can study the evolution over time of the author’s keywords usages.

As previously mentioned, this section is not intended to cover a systematic literature review but rather to illustrate some of the potential uses of powerful techniques or highlight some of the interesting questions that can be addressed. With a data set of 60,500 references, we have access to a wealth of information. Combined with the state of the technological development we have access to today, multiple questions can be answered. To go further, we could imagine analyzing document cocitations or reference burst detections [17].

## Discussion

### Further Developments

Recent developments in computing power, as well as data accessibility, offer new tools to develop policies to promote new capabilities or enhance existing skills as a way to encourage the further coevolution of new capabilities, echoing ideas put forward by Hirschman [18] more than 50 years ago. The difference is that now researchers, policy makers, and business analysts can analyze them in practice. It is capitalizing on the principles from the “wisdom of crowds” [19-22].

In this global pandemic, knowledge sharing and open data can have an impact on the solutions as well as the pace to discover the answers. With such a package, the easy dissemination through such an integrated workflow and low-level pipeline of tools may also help public policies. It allows the use of research evidence in health policy making [23].

Developing easy access to data and data modelization is of great importance for evidence-based policy making. In the past, there were lots of areas in public policy making where data were not accessible. As a result, decisions were made on assumptions coming from theoretical foundations or benchmarks from other sources. In our day and age, with more and more access to data across the world, being open data initiatives or not, evidence-based decisions are more and more possible. Numerous authors have demonstrated the role of data in informing better evidence-based policies [23-26].

This R package is updated daily when it comes to collecting the references and their metadata, and it will also be updated regularly to propose different use cases and new functionalities. We will update the modeling contribution of the package. For instance, we will integrate some of the *bibliometrix* package’s functions directly in our package to ease the scientometrist’s workflow. We will also include some models for network analysis and natural language processing–based studies.

### Conclusion

In these trying times, we believe that working with reproducible research principles based on open science, disseminating scientific information, providing easy access to scientific production on this particular issue, and offering a rapid integration in researchers’ workflows may help save time in this race against the virus, notably in terms of public health. In this context, we believe the bibliometric packages made available by research institutions, nongovernmental organizations, or individual researchers complement the other data packages and help provide a more comprehensive understanding of the pandemics. One of the objectives is to reduce “the barrier for researchers and public health officials in obtaining comprehensive, up-to-date data on this ongoing outbreak. With this package, epidemiologists and other scientists can directly access data from four sources, facilitating mathematical modelling and forecasting of the COVID-19 outbreak” [1].

This package aims at providing this easy access and integration in a researcher’s workflow. It is specially designed to collect data and generate a data frame compatible with the *bibliometrix* package [11]. Such data sets may facilitate access to the right information. Moreover, the use of massive data sets crossed with robust data analyses may foster multidisciplinary perspectives, raising new questions and providing new answers [27-29]. Classification techniques can be used to go through the large volume of references and allow researchers to save time on this part of their research. Network analysis can be used to filter the data set. Text mining techniques can also help researchers calculate similarity indices and help them focus on the parts of the literature that are relevant for their research.

The package collects references that are interesting, for the most part, for the medical domain and allows multidisciplinary perspectives on this data set. It could be interesting to get views from other disciplines, for instance, mathematics, computer science, political science, economics, and environmental science. This is the result of the emergency in which humanity finds itself right now. We could also envisage later to add references from other disciplines such as social sciences and augment, or open, the perspectives on the issue. Not only would we benefit from a multidisciplinary perspective through the methodology dimension, as the goal is with our EpiBibR package, but we would also benefit from the multidisciplinary perspectives through the ontological concepts and theories of these added domains.

## Abbreviations

AI: artificial intelligence
CDC: Centers for Disease Control and Prevention
COVID-19: coronavirus disease
MERS-CoV: Middle East respiratory syndrome–related coronavirus
NLM: National Library of Medicine
SARS-CoV: severe acute respiratory syndrome–related coronavirus
WHO: World Health Organization
2019-nCoV: novel coronavirus
